# RCSB Protein Data Bank: Architectural Advances Towards Integrated Searching and Efficient Access to Macromolecular Structure Data from the PDB Archive

**DOI:** 10.1016/j.jmb.2020.11.003

**Published:** 2020-11-10

**Authors:** Yana Rose, Jose M. Duarte, Robert Lowe, Joan Segura, Chunxiao Bi, Charmi Bhikadiya, Li Chen, Alexander S. Rose, Sebastian Bittrich, Stephen K. Burley, John D. Westbrook

**Affiliations:** 1 - Research Collaboratory for Structural Bioinformatics Protein Data Bank, Rutgers, The State University of New Jersey, Piscataway, NJ 08854, USA; 2 - Institute for Quantitative Biomedicine, Rutgers, The State University of New Jersey, Piscataway, NJ 08854, USA; 3 - Cancer Institute of New Jersey, Rutgers, The State University of New Jersey, New Brunswick, NJ 08901, USA; 4 - Research Collaboratory for Structural Bioinformatics Protein Data Bank, San Diego Supercomputer Center, University of California, La Jolla, CA 92093, USA; 5 - Department of Chemistry and Chemical Biology, Rutgers, The State University of New Jersey, Piscataway, NJ 08854, USA

**Keywords:** structural biology, databases, computer architecture, FAIR principles

## Abstract

The US Research Collaboratory for Structural Bioinformatics Protein Data Bank (RCSB PDB) serves many millions of unique users worldwide by delivering experimentally-determined 3D structures of biomolecules integrated with >40 external data resources via RCSB.org, application programming interfaces (APIs), and FTP downloads. Herein, we present the architectural redesign of RCSB PDB data delivery services that build on existing PDBx/mmCIF data schemas. New data access APIs (data.rcsb.org) enable efficient delivery of all PDB archive data. A novel GraphQL-based API provides flexible, declarative data retrieval along with a simple-to-use REST API. A powerful new search system (search.rcsb.org) seamlessly integrates heterogeneous types of searches across the PDB archive. Searches may combine text attributes, protein or nucleic acid sequences, small-molecule chemical descriptors, 3D macromolecular shapes, and sequence motifs. The new RCSB.org architecture adheres to the FAIR Principles, empowering users to address a wide array of research problems in fundamental biology, biomedicine, biotechnology, bioengineering, and bioenergy.

## Introduction

Established in 1971 as the first open-access, digital data resource in biology with just seven protein structures, the Protein Data Bank (PDB)^1^ is universally regarded as a fundamental and core data resource essential to the basic and applied research in the life-sciences, fundamental biology, biomedicine, biotechnology, bioengineering, and energy communities. Now in its 50th year of continuous operation, the PDB serves as the singular global repository for 3D structural information, making >170,000 experimentally determined structures of proteins, DNA, RNA, and their complexes with drugs and/or other small molecules freely available without usage limitations.

Since 2003, the PDB has been managed jointly by the Worldwide Protein Data Bank (wwPDB) partnership,^2,3^ (including US-funded Research Collaboratory for Structural Bioinformatics Protein Data Bank or RCSB PDB,^4,5^ Protein Data Bank in Europe,^6^ Protein Data Bank Japan,^7^ and the Biological Magnetic Resonance Data Bank.^8^ wwPDB partners provide global deposition-validation-biocuration services to guarantee that archived structures are as complete as possible and receive consistent validation and expert biocuration.^9–11^ In addition to wwPDB data acquisition and archiving, the RCSB PDB provides a variety of data delivery services packaging the primary archival data integrated with additional content from >40 leading biological and life science resources. These services include tools enabling data search, browsing, custom report generation, visualization, and analyses tailored for both programmatic and web interactive users. Collectively these activities and services strengthen our enduring commitment to the **FAIR** Principles of **F**indability-**A**ccessibility-**I**nteroperability-**R**eusability.^12^

The RCSB PDB (hereafter RCSB) assumed responsibility for the PDB within the US in 1999. Since then, RCSB data delivery services have undergone substantive design changes. Redesigns have been motivated by proactive efforts to address challenges arising from new primary data entering the PDB archive,^3^ growth in related community domain data resources, and dramatic growth and broadening of the diverse array of PDB data.^13–15^ Among the challenges coming from new primary data sources are growing numbers of depositions, increasing size and molecular complexity of deposited structures, and the rapidly evolving technology landscape in structural and computational biology. External resources targeted for data integration in the biological and life sciences have grown significantly in both number and scale, making it ever more demanding to maintain data correspondence and mapping information for interoperability across and beyond this bioscience data ecosystem. In addition to challenges arising from primary and integrated data, the growing capacity, performance, and feature requirements coming from both web-based interactive and programmatic RCSB users represent ongoing drivers for data delivery service redesign. Concurrently, regular improvements have been necessary to maintain IT infrastructure implemented using contemporary tools and modern software engineering best-practices. The April 2020 release of new RCSB data services represents the most significant upgrade in overall design to date, implementing sweeping changes in both data and software architecture. The following sections present features and capabilities of this new RCSB service architecture.

## Methods

### Architecture overview

**Figure 1.**
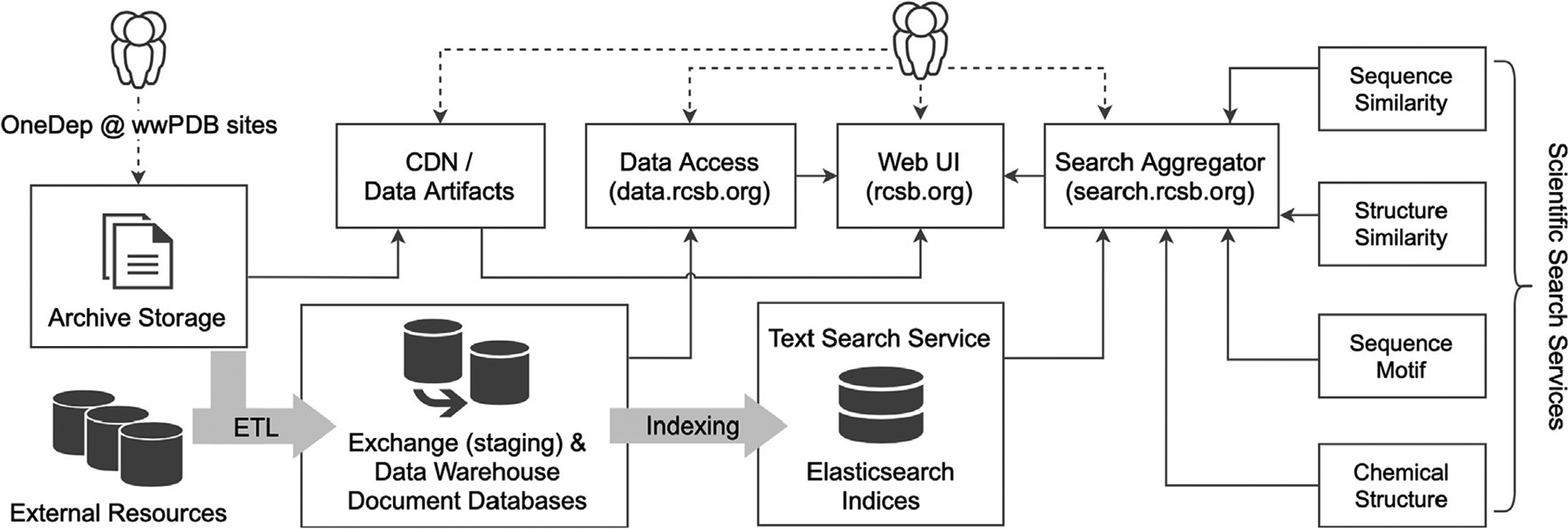
Data management and delivery system underpinning the new RCSB architecture.

The new RCSB data and search service architecture is illustrated in Figure 1. With this implementation, we transitioned from a large feature-rich multi-purpose web application to a modern architecture within which services are delivered by a collection of loosely coupled collaborating applications each with narrow, well-defined responsibilities. A major software overhaul decomposed the previous implementation into small single-purpose services, with logical service boundaries, and well-defined connecting APIs.

Decomposition yielded services supporting both search and data access functions. New back-end services are individually responsible for searches of text and structured attributes, sequence similarity, sequence motifs, structure similarity, and chemical similarity. These search services are orchestrated by an aggregation application responsible for dispatching search tasks to appropriate back-end search services and combining results therefrom.

Deposited primary data integrated with external data supporting search and data access services are managed in a data warehouse, the document store populated with structured data that acts as the authoritative source of content in the architecture. Structured data are indexed for text and attribute searches. Raw data artifacts are delivered by a content delivery network (CDN) service. Data access services (data.rcsb.org) are provided through both REpresentational State Transfer (REST)^16^ and GraphQL^17^ APIs.

Data flow along the pipeline is illustrated in Figure 1. Data from the regional wwPDB deposition sites are added to the PDB archive and shared with the wwPDB partners on a fixed weekly schedule. The jointly managed archive provides the source of primary PDB data for the RCSB data delivery pipeline. The ETL (*i.e*., Extract, Transform, Load) and service deployment operations supporting this update workflow are orchestrated by a Luigi workflow management agent (github.com/spotify/luigi). The search aggregator service (search.rcsb.org) and data access service (data.rcsb.org) provide entry points for search and access operations shared by both the RCSB web front-end application (rcsb.org) and public-facing programmatic web services. In this new architecture, the RCSB.org website front-end has adopted a modern and extensible front-end web framework, while retaining the familiar look and feel of the resource.

### Data and schema

An important feature of overall data management in the new RCSB architecture (Figure 1) is continuity in metadata management spanning the full data lifecycle. Use of metadata starts at the beginning of the pipeline with acquisition of depositor-provided information, followed by validation and biocuration of these data by the wwPDB OneDep system.^9^ To ensure consistency and extensibility in all of its data process operations, OneDep and its supporting tools rely on a digital metadata dictionary as the authoritative reference source for information about PDB archive data. This metadata reference dictionary is a product of the PDBx macromolecular Crystallographic Information Framework (mmCIF),^18–23^ which evolved from standardization efforts of the International Union of Crystallography that began in the 1990s.^24^ In 2014, PDBx/mmCIF became the internationally-recognized metadata standard for the PDB archive^25^ and in 2019 PDBx/mmCIF was required for deposition of atomic coordinates generated using macromolecular crystallography (MX).^26^

The PDBx/mmCIF data model is highly structured, defining a rich collection of biological, molecular, chemical, structural data, and data quality features. PDBx/mmCIF is a dynamic data model that grows continuously with technology evolution and methodological advances in structural biology. PDB chemical and molecular reference data^27,28^ are also managed within the PDBx/mmCIF schema. This data model further provides metadata required to perform precise semantic alignment of data integrated from external data resources.

The organization of the PDBx/mmCIF data model^20–23,26,29–30^ is tabular, allowing facile management of data represented in this framework using relational database tools. In addition to providing data content specification, the PDBx/mmCIF metadata framework contains data typing, data provenance, validation, and organizational details required for automated checking of data consistency. The wwPDB partners in collaboration with community domain experts (wwPDB PDBx/mmCIF Working Group) coordinate development and maintenance of the PDBx/mmCIF dictionary. Working Group deliberations and data dictionary content are published on the GitHub platform (github.com/pdbxmmcifwg) and a data portal site (mmcif.wwpdb.org), respectively.

Metadata inform the next step of the new data delivery workflow (Figure 1), in which the largely tabular archival primary data is projected onto a document hierarchy that reflects the underlying macromolecular structural hierarchy. Biological macromolecules have a natural hierarchy, building from units of different granularity extending from atoms to polymer components (*e.g*., amino acids) to polymer chains to assemblies of interacting polymer chain macromolecules and ligands. The document data model is well-suited to handling the hierarchical data representation for macromolecular structures. Data organization is formalized in a document schema within which features describing a particular level in the macromolecular hierarchy are grouped into document collections. The current document schema includes collections describing the following: deposited data (or entry); macromolecular assemblies generated from the entry; decomposition of the entry in terms of distinct polymer, branched (*e.g*., oligosaccharide), and non-polymer molecular entities (*e.g*., small-molecule ligands such as co-factos and enzyme inhibitors); and the observed atomic structure instances of these molecular entities in the deposited data set. The schema does not include raw atomic-level coordinates. Information about atomic-level coordinate data including counting statistics, completeness, and a range of experimental and geometrical data quality metrics are summarized within the molecular hierarchy. The document schema includes a subset of available primary (PDBx/mmCIF) data content that is well-populated across the entire archive.

Operationally, transformation to the document organization takes place as the archival data products of the OneDep system are loaded into an intermediate staging document store (Figure 1: Exchange DB). This step requires both data and schema transformations with the latter being encoded in a standard JSON (JavaScript Object Notation) schema representation (json-schema. org) with some local RCSB extensions. Some essential data integration tasks closely tied to the primary data and chemical reference data are also performed at this stage. These ETL operations include updates to reference sequence database correspondences plus reference database correspondences for small molecules in PDB chemical and molecular reference data.

The Data Warehouse (DW, Figure 1) provides the authoritative source of data for all RCSB data access and search services. Primary archival data loaded into the staging database (Exchange DB) with data from external resources processed by local ETL operations are federated into the DW document store. The DW document store is in turn responsible for supporting the data access needs of the RCSB.org website and the public-facing RCSB programmatic data access API services.

A document organization was chosen for the new DW because this approach most closely resembles the predominant data access patterns employed in assembling content for the RCSB.org website and in delivering programmatic web services (data.rcsb.org). Having the data organized in this readily accessible document format improves retrieval performance and avoids computationally expensive JOIN operations on normalized tabular data that were required with our previous relational database architecture.

The DW data schema extends the primary data schema used in the staging database (Exchange DB) with additional annotations coming from external resources. Addition of these annotations to the data schema allows the origin of each annotation to be clearly defined. These schema revisions and extensions within the new architecture are managed and automatically deployed through a GitHub (github.org) version control workflow.

In the new architecture, the main communication medium for data exchange for the different service APIs is JSON, making JSON Schema a convenient format choice to store and exchange schema information. The DW data schema, encoded using JSON Schema language, includes attribute descriptions, examples, and validation keywords such as data type, controlled vocabularies, and boundary values. The DW is hosted in a MongoDB (mongodb.com) document-oriented database, which supports document validation using a flavor of JSON schema.

Data schema represent essential vehicles for sharing data specifications among different services. Knowledge of the data schema is essential in a modular architecture composed of many collaborating services wherein ensuring data integrity and consistency between the services is critically important. Central consolidation of data schema in a technology-agnostic format allows this information to be shared among components of the data management and delivery system.

**Figure 2.**
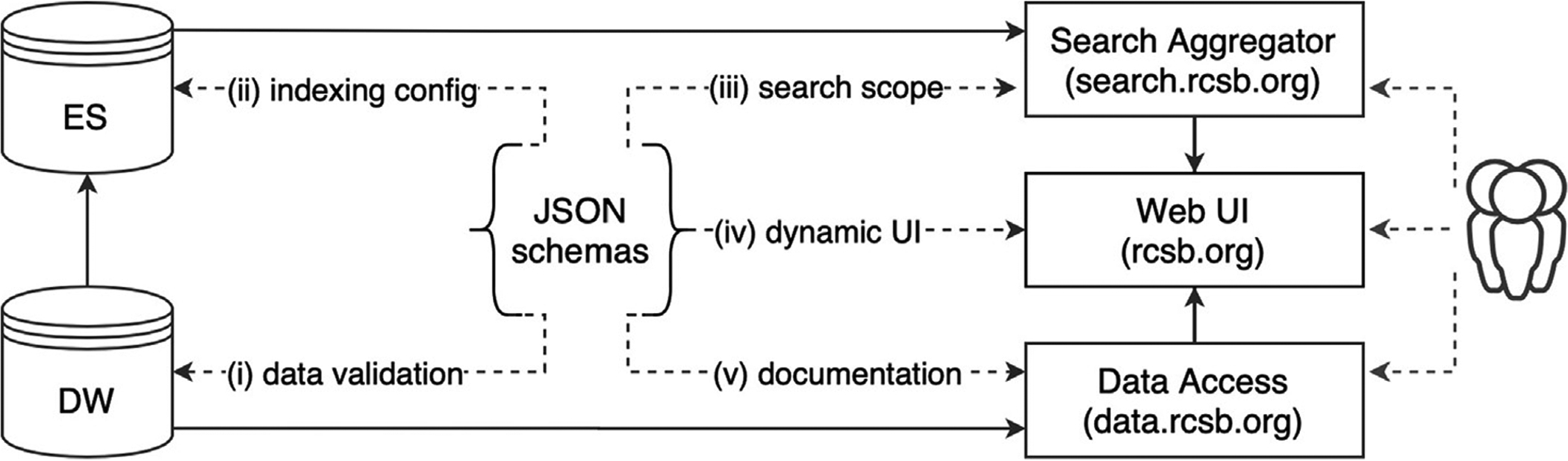
Schema usage by different components of the data management and delivery system.

Figure 2 illustrates how the JSON schema is used to establish contracts between services in our data management and delivery system: (i) JSON schema validation constraints are used by ETL processes to check data before loading the DW; (ii) search indexing processes use JSON schema data types to automatically create an indexing configuration; (iii) the Search Aggregator service uses JSON schema metadata describing searchable attributes and possible search operations to validate search requests; (iv) the front-end RCSB.org module uses the same metadata to dynamically construct the RCSB.org Advanced Search query builder, and supporting pages of documentation describing searchable attributes; and (v) the data access module uses metadata type details to enable type-safe data parsing and automatic documentation generation for the data access services.

### Data access services

The data access service (data.rcsb.org) provides the gateway to the DW datastore (Figure 1). This service is implemented as a lightweight application delivering both GraphQL and traditional REST-based APIs over an HTTP/S protocol.

The GraphQL query language provides composable access to the full range of content within the DW, allowing users to craft custom requests for subsets of data that match particular needs. The new GraphQL API was implemented using an open-source SPQR (Schema Publisher & Query Resolver) Java library (github.com/leangen/graphql-spqr). The scope of content available to GraphQL is defined in a GraphQL schema, which is automatically generated from our JSON schemas (Figure 2). Programmatic GraphQL API requests are served through a single URL/endpoint (data.rcsb.org/graphql). In addition to the API, the GraphQL service provides an interactive browser-based user interface (UI), *GraphiQL* (data.rcsb.org/graphql) that facilitates composing, validating, and testing GraphQL API queries. This UI also exposes the rich documentation defined in the data schema.

The new REST API is implemented using the open-source JAX-RS support in the Jersey Java framework (eclipse-ee4j.github.io/jersey). The defined collection of URLs/endpoints are organized to reflect the underlying data hierarchy of the new data architecture. For example, the REST API entity group provides access to features of distinct polymer, branched entity (*e.g*., oligosaccharides), and non-polymer entity data. REST API service responses are delivered as JSON payloads. REST endpoints documentation (data.rcsb.org/redoc) including input parameters and output data schema is generated as the OpenAPI specification (www.openapis.org) and rendered using ReDocUI tool (redoc.ly).

### CDN services

Static data assets are delivered through content delivery network services (Figure 1: CDN). The file service (files.rcsb.org) provides access to data hosted in the PDB FTP repository. This body of information includes primary archival data files containing atomic coordinates and files containing supporting experimental data. Macromolecular structure images (*e.g*., assemblies, structures, sub-structures), small-molecule chemical diagrams, carbohydrate SNFG^31^ diagrams, and other static content used by the RCSB.org website are served by the CDN service (cdn.rcsb.org).

Specialized services have been developed to efficiently deliver large data artifacts to web-based molecular graphics and analysis tools. These services, built on the open-source Mol* library,^32^ enable interactive manipulation and analysis of the largest macromolecular assemblies, 3D Electron Microscopy (3DEM) map volumes, and electron density maps from MX in the PDB repository. The model service (models.rcsb.org) delivers atomic coordinates together with the annotations in the primary data files in a compressed binary CIF encoding (BCIF).^33^ Structure data can be served at different levels of granularity (*e.g*., assembly, polymer chain, ligand), and ligand data may also be delivered in popular chemical informatics formats (*e.g*., SDF, MOL, MOL2). The volume service (maps.rcsb.org) provides access to volumetric data from MX electron density and 3DEM volume maps. This service can also deliver volume subsets and optionally downsample the volumetric data to reduce data transfer bandwidth requirements.

### Search aggregator service

The responsibilities of the search aggregator service (Figure 1: Search Aggregator) include: (i) providing the single-entry point for all search operations, (ii) routing requests to the appropriate underlying search services for processing, and (iii) applying basic Boolean logic operations to combine the multiple search results.

This service enables searches across elements of macromolecular structure data at different levels of granularity. A common example involves searching for macromolecular assemblies containing a protein similar to a target sequence bound to a ligand similar to a drug target. Here, the results from the two search modes, for small molecules and protein sequences, are first merged at the granularity of the assembly result set type and then combined with a Boolean AND operator.

The aggregator service provides a uniform API layer that abstracts details required to merge and combine the results of the underlying search services. To support this API, a custom domain-specific language (DSL) has been developed to describe search queries. Queries in this custom DSL are represented as a graph. Nodes in this graph can represent either individual or group searches. A simple terminal node describes a single search operation (*e.g*., an attribute-based search or sequence search). A group node combines multiple nodes with a Boolean combination operation. Nodes in the query graph may be arbitrarily nested allowing for the construction of complex search patterns. While the introduction of our custom DSL added some additional software development effort required to build the search aggregator service, it has been more than offset by the greater flexibility and extensibility that the DSL has afforded. In particular, the custom DSL provides a simple abstraction for query construction that encapsulates the implementation differences in the underlying search services.

The search aggregator application is implemented as a lightweight stateless REST-style web service. The service endpoint (search.rcsb.org/rcsbsearch/v1/query) provides a JSON-based API implemented using the Java Jersey framework (eclipse-ee4j.github.io/jersey). The API accepts HTTP/S GET and POST and returns a JSON response.

### Search services

The new architecture (Figure 1) implements searches for text and structured attributes, sequence similarity, sequence motifs, structure similarity, and chemical similarity as independent services all orchestrated by the search aggregator.

The Attribute and Text Search service enable composable queries on the content of the DW document store. This service is built on the open-source Elasticsearch search engine (V7: https://www.elastic.co). Elasticsearch transforms JSON documents from the DW into *Inverted Indices* optimized for type-specific structured attribute searches or unstructured text searches. Structured attribute queries enable matching numbers, Boolean values, dates, and exact text values. Keywords and phrases can be matched as unstructured text. Search results are returned as identifiers for DW documents at the desired granularity in the DW data model. Matching documents are returned with an internally calculated relevance score allowing rank ordering of results by significance.

The Sequence Similarity Search service enables performant queries for protein, DNA, and RNA one-letter-code sequences in the PDB archive by employing the sequence comparison tool MMseqs2.^34,35^ For each matching sequence, the service returns a unique identifier for the PDB polymer sequence, matching scores (*e.g*., sequence identity, E-value, and bit-score), and the residue boundary positions of the matching sequence.

The Sequence Motif Search service enables queries on protein or nucleic acid polymer sequences, using three different types of input format simple one-letter-code sequence patterns, regular expressions of one-letter-code patterns, and PROSITE^36^ patterns. The service returns PDB polymer entity identifiers and residue boundaries for matches in the sequences.

The Structure Similarity Search service enables queries for global similarity in spatial arrangements of macromolecules and macromolecular assemblies. The service employs a computationally-efficient BioZernike method developed by RCSB.^37^ To perform fast shape comparisons the method uses pre-calculated rotationally invariant descriptors of the volumetric and geometric representations of each 3D shape. The service outputs identifiers of the matching structural elements along with similarity scores calculated for volumetric and geometric descriptors.

The Chemical Search service enables queries of small-molecule constituents of PDB IDs or structures, based on chemical formula and chemical structure. Both molecular formula and formula range searches are supported. Queries for matching and similar chemical structures can be performed using SMILES^38^ and InChI^39^ descriptors as search targets. Graph and chemical finger-print searches are implemented using tools from the OpenEye Chemical Toolkit (www.eyesopen.com/oechem-tk).

### Infrastructure support

The new architecture (Figure 1) is deployed in a private cloud environment built on the open-source OpenStack cloud platform. Local tools have been developed using the OpenStack API for creating and deploying custom cloud virtual machine instances. Customizable instance configurations enable efficient management of overall physical resources which can flexibly adapt to changes in user demands. The RCSB manages cloud resources in geo-redundant data centers located on the campuses of the University of California, San Diego and Rutgers, The State University of New Jersey.

## Results and discussion

To illustrate the power of the new search and data access capabilities, we present the example of finding 3D structures for a particular subset of ligand-protein complexes related to SARS-CoV-2 and then accessing a wide range of metadata associated with the matching structures. Analogous examples are also available^40^ describing how these capabilities have been used to develop new search and data access tools for the RCSB PDB web resource (RCSB.org).

**Figure 3.**
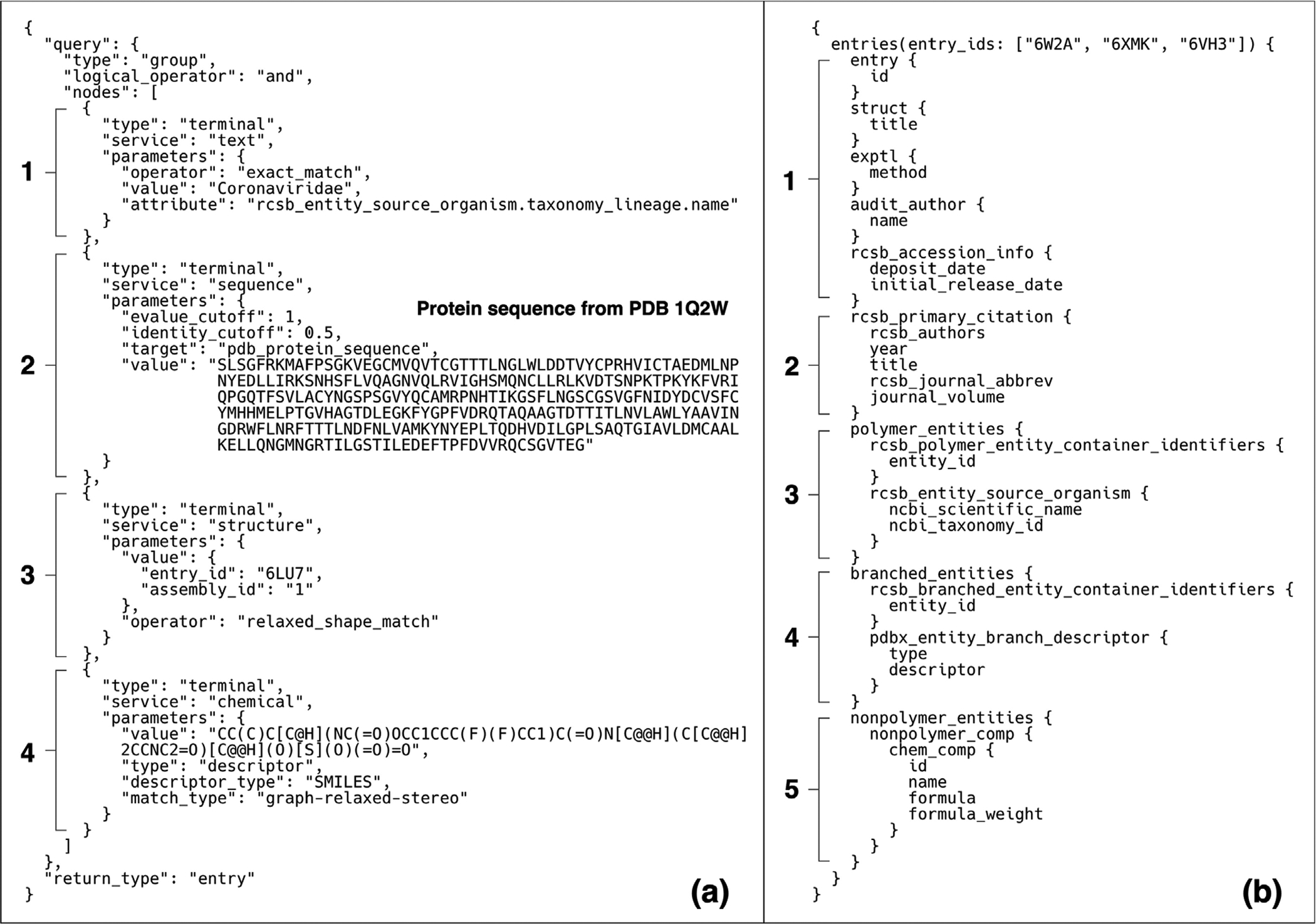
Example search and data access queries: (a) query that combines text (1), sequence (2), structure shape (3), and chemical similarity (4) searches; (b) GraphQL API query including essential entry details (1–2), information details of the macromolecular entity data hierarchy (2–4) and small-molecules (5).

The search service API request for this example is depicted in Figure 3(a). It combines full-text (a1), sequence (a2), structure (a3), and chemical similarity (a4) search modes. The text search targets structures of the *Coronaviridae* family taxonomy. The sequence similarity search (a2) targets the sequence of the SARS-CoV Nsp5 domain (PDB ID 1Q2W)^41^ with a comparison threshold of 50% sequence identity. The structure similarity search targets the shape of the first biological assembly similar in PDB ID 6LU7 (SARS-CoV-2 Nsp5).^42^ The chemical similarity search targets the 3C-like protease inhibitor 7J,^43^ represented as a SMILES chemical descriptor. At the time of writing, performing each of these searches and applying a Boolean AND operation to select the only common results yields 3 structures satisfying all of the search criteria (PDB IDs: 6W2A, 6XMK, 6VH3).^43^ This example showcases how accessing four different search services is achieved through a single API request to the Search Aggregator service (Figure 1).

The companion data access API request is shown in Figure 3(b) illustrating the GraphQL API service request to fetch details spanning the DW schema hierarch. This query includes entry-level attributes such as the entry title, experimental methods, depositors, deposition and release dates (b1); primary citation data (b2); taxonomy details for polymeric entities (b3), descriptive information for branched entities (*e.g*., oligosaccharides) (b4); and names, formulae, and formula weight for small-molecule ligands (b5). The attributes in this example can also be accessed using the REST API; however, multiple different requests are required to retrieve the same data. The advantage of the GraphQL API is the ability to craft a single API request for all of the desired data content.

A key objective of our architectural redesign has been improving the overall FAIR-ness of the RCSB data delivery services. As the preceding example demonstrates, improvements in search and data access services significantly advance the Findability and Accessibility of the PDB structure data. Consolidation of data schema and adoption of community standards such as OpenAPI, JSON Schema, and JSON for API data exchange collectively improve both data Interoperability and Reusability.

We hope the outcome of the architectural redesign will enhance operational efficiencies, improve deployment scalability, reduce the time for the rollout of new features and bug fixes, and enable more proactive targeted monitoring of service health. The architectural redesign is also expected to pave the way for more economical future deployments in publicly hosted cloud resources.

## Supplementary Material

1

## Abbreviations used:

3DEM: Three-dimensional Electron Microscopy
API: Application Programming interface
FAIR: Findability, Accessibility, Interoperability, and Reusability
MX: Macromolecular Crystallography
REST: REpresentational State Transfer
