# Supplementary material for: RCSB Protein Data Bank: Architectural Advances Towards Integrated Searching and Efficient Access to Macromolecular Structure Data from the PDB Archive
Source: J Mol Biol. Author manuscript; Available in PMC 2022 May 11. (PMC9093041; doi:10.1016/j.jmb.2020.11.003)
Supplement: 1 [file NIHMS1804367-supplement-1.pdf]

Supplemental Data

RCSB Protein Data Bank: architectural advances towards integrated searching and efficient access to macromolecular structure data from the PDB archive.

JSON Schema Name	URL (extension data.rcsb.org/rest/v1/schema)
Entry	/entry
Assembly	/assembly
Polymer Entity	/polymer_entity
Branched Entity	/branched_entity
Non-Polymer Entity	/nonpolymer_entity
Polymer Entity Instance	/polymer_entity_instance
Branched Entity Instance	/branched_entity_instance
Non-Polymer Entity Instance	/nonpolymer_entity_instance
Chemical Component/BIRD	/chem_comp
PubMed	/pubmed
UniProt	/uniprot
DrugBank	/drugbank

Table 1. RCSB PDB JSON data schema.

Data API Endpoint	URL Scheme (after data.rcsb.org/rest/v1/core)
Entry	/entry/{entry_id}
Assembly	/{entry_id}/{assembly_id}
Polymer Entity	/polymer_entity/{entry_id}/{entity_id}
Branched Entity	/polymer_entity/{entry_id}/{entity_id}
Non-Polymer Entity	/nonpolymer_entity/{entry_id}/{entity_id}
Polymer Entity Instance	/polymer_entity_instance/{entry_id}/{asym_id}
Branched Entity Instance	/branched_entity_instance/{entry_id}/{asym_id}
Non-Polymer Entity Instance	/nonpolymer_entity_instance/{entry_id}/{asym_id}
Chemical Component/BIRD	/chemcomp/{comp_id}
PubMed	/pubmed/{entry_id}
UniProt	/uniprot/{entry_id}/{entity_id}
DrugBank	/drugbank/{comp_id}

Table 2. REST API endpoints supported at data.rcsb.org.

Data API GraphQL Query Examples	URL
Entry	data.rcsb.org/#gql-example-1
Primary Citation	data.rcsb.org/#gql-example-2
Polymer Entity	data.rcsb.org/#gql-example-3
Polymer Entity Instance	data.rcsb.org/#gql-example-4
Branched Entity	data.rcsb.org/#gql-example-5

Table 3. GraphQL API examples illustrating data access at specific levels of the molecular hierarchy.

Search API Query Examples	URL
Protein Sequence Search	search.rcsb.org/#search-example-3
Sequence Motif Search	search.rcsb.org/#search-example-6
3D-shape Search	search.rcsb.org/#search-example-4
Chemical Similarity Search	search.rcsb.org/#search-example-7
Combined Search Services	search.rcsb.org/#search-example-10

Table 4. Search API examples for sequence, sequence motif, 3D shape, chemical search and combined multi-mode search operations.
